# miR-195-5p alleviates acute kidney injury through repression of inflammation and oxidative stress by targeting vascular endothelial growth factor A

**DOI:** 10.18632/aging.103160

**Published:** 2020-06-03

**Authors:** Yong Xu, Wei Jiang, Lili Zhong, Hailun Li, Lin Bai, Xiaoling Chen, Yongtao Lin, Donghui Zheng

**Affiliations:** 1 Department of Nephrology, Huai'an Second People's Hospital and The Affiliated Huai'an Hospital of Xuzhou Medical University, Huai'an, China; 2 Department of Nephrology, Siyang Hospital of Traditional Chinese Medicine, Suqian, China

**Keywords:** acute kidney injury, miR-195-5p, VEGFA, inflammation, oxidative stress

## Abstract

Acute kidney injury (AKI) is a common renal dysfunction. Renal ischemia-reperfusion (I/R) injury contributes to AKI progression. The microRNA miR-195-5p can act as a crucial tumor inhibitor in various cancers. However, the potential biological effects of miR-195-5p on AKI are not well-understood. We found that miR-195-5p levels were decreased in the serum samples of patients with AKI. Next, we determined miR-195-5p expression in the renal tissues of the rats and found that it was downregulated. Renal function was evaluated and confirmed using blood urea nitrogen and serum Cr levels. In parallel, the hypoxia-induced NRK-52E cell model was employed, and miR-195-5p was found to be markedly reduced under hypoxic conditions. Furthermore, miR-195-5p was modulated in NRK-52E cells. miR-195-5p induced NRK-52E cell proliferation and protected NRK-52E cells against hypoxia-triggered apoptosis. In an I/R mouse model, miR-195-5p alleviated renal injury triggered by I/R. In addition, oxidative stress and inflammatory factor concentrations were assessed using ELISA. The results showed that miR-195-5p mimicked attenuated oxidative stress induced by I/R injury and downregulated the protein expression of inflammatory factors. Moreover, we identified that vascular endothelial growth factor A (VEGFA) was a target gene of miR-195-5p, which could negatively regulate VEGFA expression in vitro. Inhibitors of miR-195-5p subsequently contributed to renal injury, which was reversed by VEGFA loss. In conclusion, miR-195-5p may repress AKI by targeting VEGFA.

## INTRODUCTION

Acute kidney injury (AKI) is a complex disease that involves a decrease in the glomerular filtration rate (GFR). Ischemia and exposure to nephron toxicants can contribute to AKI [1]. Ischemia-reperfusion (I/R) injury is a tissue injury that can result from blood reperfusion, and renal I/R injury is a common reason for AKI progression [2]. Increasing data has revealed that ROS generation and inflammatory factors can result in renal tissue damage [3].

MicroRNAs are small RNAs that can repress gene expression via mRNA degradation or translation repression [4–6]. miRNAs play crucial roles in kidney physiological functions [7–8]. For example, in lupus nephritis, miR-150 can induce fibrosis of renal tissues by targeting SOCS1 [9]. miR 30a 5p can function as a tumor suppressor in renal cell carcinoma [10]. In addition, miR 181 can play an inhibitory role during renal fibrosis by attenuating profibrotic marker expression [11]. IR injury can play a major role in AKI. For instance, miR-125b can act as a novel biomarker of renal I/R injury [12]. miR-146 can prevent injury in I/R by targeting IGSF1 and exert a renal protective effect [13]. In addition, miR-194 overexpression can reduce hypoxia/reperfusion-triggered HK-2 cell injury by regulating Rheb [14].

miR-195-5p belongs to the microRNA-15a/b/16/195/497 family [15]. miR-195-5p has been reported in many cancers and can act as a tumor suppressor. For example, miR-195 represses breast cancer tumor progression by regulating IRS1 [16]. miR-195 suppresses prostate carcinoma progression by directly targeting BCOX1 [17]. miR-195 can depress hepatocellular carcinoma progression by targeting FGF2 [18]. However, the potential biological effects of miR-195-5p on AKI are not well understood. Here, we report that miR-195-5p was greatly reduced in AKI. Vascular endothelial growth factor A (VEGFA) was predicted as the downstream target of miR-195-5p. Therefore, we hypothesize that miR-195-5p exhibits an inhibitory role in AKI by targeting VEGFA.

## RESULTS

### miR-195-5p was downregulated in AKI

**Figure 1:**
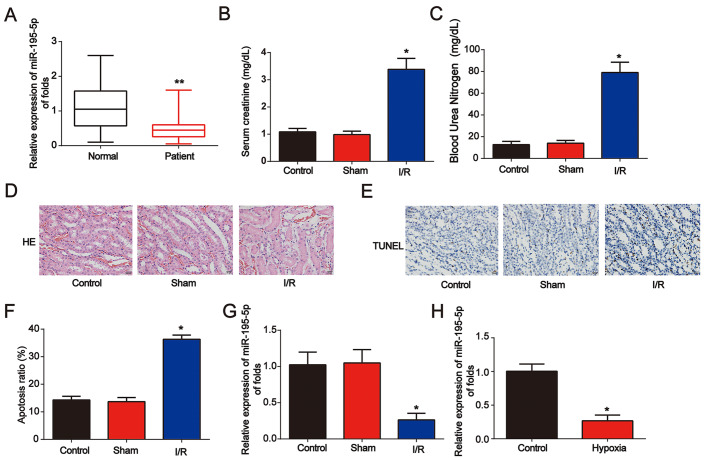
**Identification of miR-195-5p in AKI**. (**A**) Analysis of miR-195-5p in serum from healthy controls and patients with AKI. U6 served as a reference control. (**B)** Serum Cr levels in I/R rat models. (**C**) BUN levels in I/R rat models. (**D**) Representative micrographs of renal histologic findings. Scale bars = 20 μm. (**E** and **F**) Analysis of apoptosis using the TUNEL assay in renal tissues from AKI rats. (**G**) miR-195-5p expression in I/R rat models. (**H**) miR-195-5p expression in NRK-52E cells. Cells were exposed to hypoxia for 6 h. Eight rats were used in each group. Three independent experiments were performed. Error bars represent the mean ± SD of at least three independent experiments. *P < 0.05; **P < 0.01.

First, to study the effect of miR-195-5p in renal disease, serum samples from healthy controls (n = 80) and AKI patients (n = 80) were obtained. qRT-PCR was performed and miR-195-5p levels were decreased in AKI patients (Figure 1A). Then, as shown in Figure 1B and 1C, a renal I/R rat model was established, and serum Cr and blood urea nitrogen (BUN) levels were markedly increased after I/R surgery. Acute kidney injury was triggered as indicated by HE staining and TUNEL assay (Figure 1D–1F). In the renal I/R rat model, miR-195-5p was markedly increased (Figure 1G). In addition, an in vitro assay was performed. NRK-52E cells were randomly assigned into two groups: control (normoxic conditions for 6 h) and hypoxia (hypoxic conditions for 6 h). We found that miR-195-5p was inhibited after NRK-52E cells were exposed to hypoxia treatment for 6 h (Figure 1H). These data indicate that miR-195-5p is involved in AKI progression.

### The influence of miR-195-5p on NRK-52E cell proliferation and apoptosis under hypoxia in vitro

**Figure 2:**
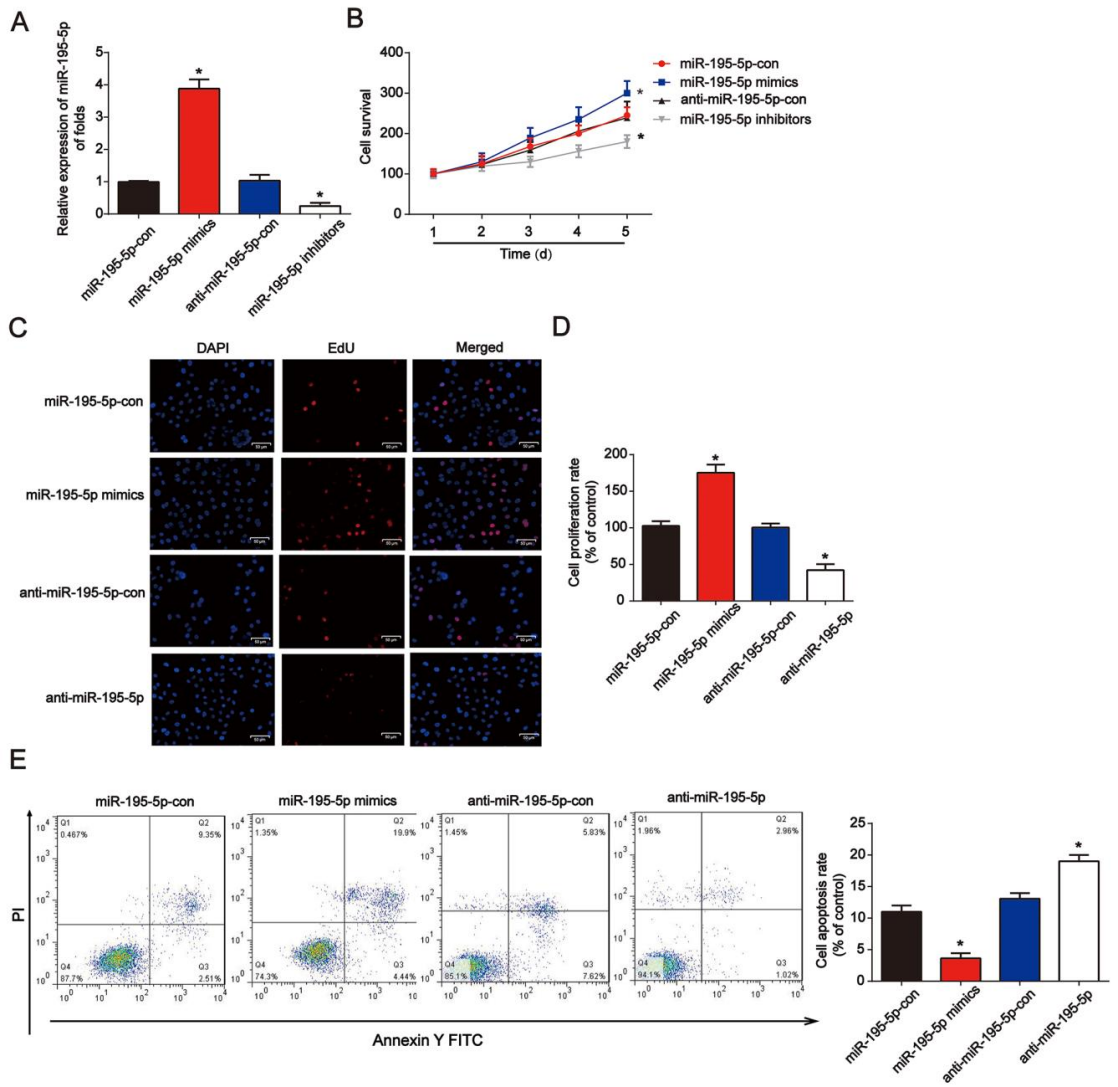
**Overexpression of miR-195-5p reduced hypoxia-induced renal injury in NRK-52E cells**. (**A**) miR-195-5p expression in NRK-52E cells transfected with miR-195-5p mimics, inhibitors, or the corresponding negative controls for 48 h. (**B**) The CCK-8 assay was carried out to test cell viability. (**C** and **D**) EdU assays were performed to detect cell proliferation. (**E**) Flow cytometry analysis was employed to test cell apoptosis. NRK-52E cells were transfected with miR-195-5p mimics or corresponding negative controls for 48 h. Three independent experiments were performed. Error bars represent the mean ± SD of at least three independent experiments. *P < 0.05.

Next, miR-195-5p mimics or inhibitors were transfected into NRK-52E cells for 48 h. qRT-PCR was used to confirm the efficiency of the miR-195-5p mimics or inhibitors in NRK-52E cells (Figure 2A). In Figure 2B, miR-195-5p mimics markedly increased cell survival, while the inhibitors reduced NRK-52E cell survival, as demonstrated by the CCK-8 assay. In addition, the EdU assay showed that NRK-52E cell proliferation was induced by miR-195-5p mimics, while repressed by miR-195-5p inhibitors (Figure 2C and 2D). Additionally, after transfection for 48 h, miR-195-5p rescued the apoptotic cells induced by hypoxia, as indicated by the flow cytometry assay (Figure 2E). miR-195-5p mimics reduced the ratio of apoptotic cells induced by hypoxia, as indicated by the flow cytometry assay. miR-195-5p inhibitors contributed to NRK-52E cell apoptosis These results indicated that miR-195-5p improved NRK-52E cell proliferation and inhibited cell apoptosis.

### miR-195-5p repressed I/R-induced renal injury in vivo

**Figure 3:**
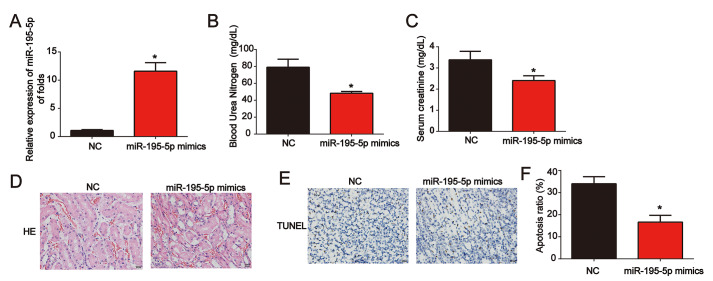
**Overexpression of miR-195-5p inhibited I/R-induced renal injury in vivo**. (**A**) miR-195-5p expression in renal tissues. (**B**) Serum Cr levels in I/R rat models transfected with miR-195-5p mimics. (**C**) BUN levels in I/R rat models transfected with miR-195-5p mimics. (**D**) Representative micrographs of renal histologic findings. Scale bars = 20 μm. (**E** and **F**) Analysis of apoptosis using the TUNEL assay in renal tissues from AKI rats. Eight rats were used in each group. Three independent experiments were performed. Error bars represent the mean ± SD of at least three independent experiments. *P < 0.05.

Moreover, miR-195-5p levels were significantly increased in renal tissues (Figure 3A). Meanwhile, serum Cr levels and BUN levels in I/R rat models transfected with miR-195-5p mimics were also reduced by miR-195-5p (Figure 3B and 3C). Representative micrographs of renal histological staining indicated that miR-195-5p repressed kidney injury (Figure 3D). Analysis of the TUNEL assay revealed that the apoptosis induced by I/R was reduced by miR-195-5p mimics (Figure 3E and 3F). These results indicated that miR-195-5p repressed I/R-triggered renal injury.

### miR-195-5p mimics reduced oxidative stress and inflammation in vivo

**Figure 4:**
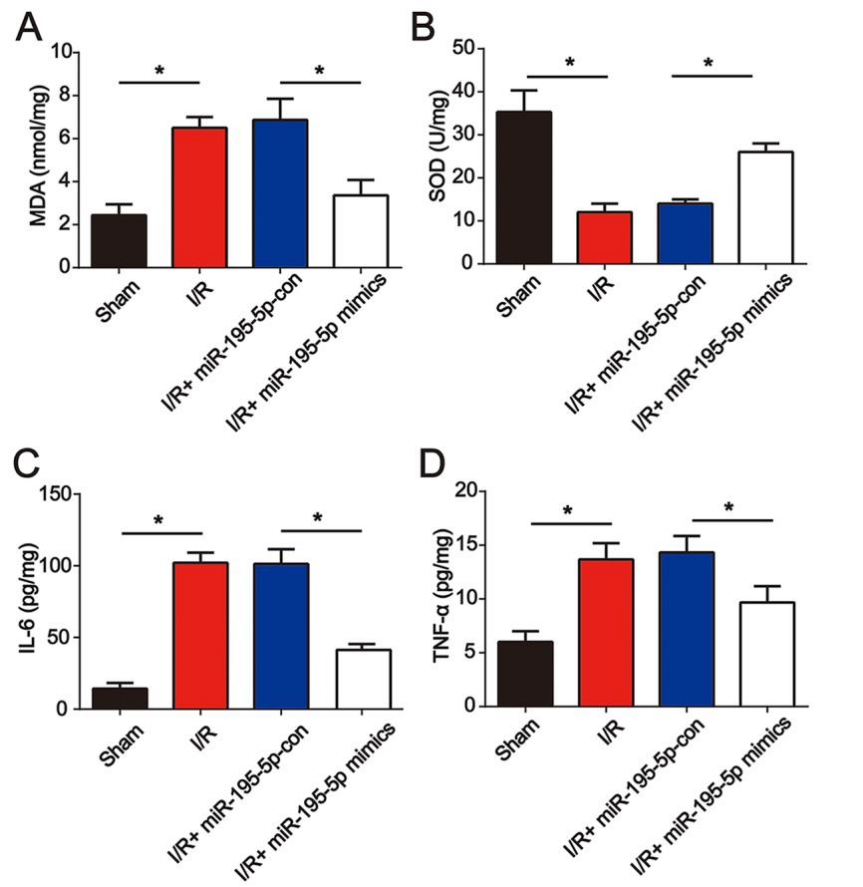
**miR-195-5p mimics attenuated renal I/R induced oxidative stress and inflammation**. miR-195-5p mimics were injected into the rat via the tail vein prior to I/R surgery. The renal tissues were collected at 24 h post I/R surgery followed by evaluation of the concentration of MDA (**A**) and SOD (**B**). IL-6 (**C**) and (**D**) TNF-α protein levels were analyzed using ELISA. Eight rats were used in each group. Three independent experiments were performed. Error bars represent the mean ± SD of at least three independent experiments. *P < 0.05.

Furthermore, before I/R surgery, miR-195-5p mimics were injected into the rat via the tail vein. We found that miR-195-5p mimic treatment repressed I/R-induced MDA and reduced SOD (Figure 4A and 4B). In addition, IL-6 and TNF-α levels were dramatically elevated at 24 h post renal I/R surgery (Figure 4C and 4D). Renal I/R-triggered cytokine production was reduced by miR-195-5p (Figure 4C and 4D). These results indicated that miR-195-5p ameliorated oxidative stress and inflammation in renal injury.

### VEGFA was a downstream target of miR-195-5p in vitro

**Figure 5:**
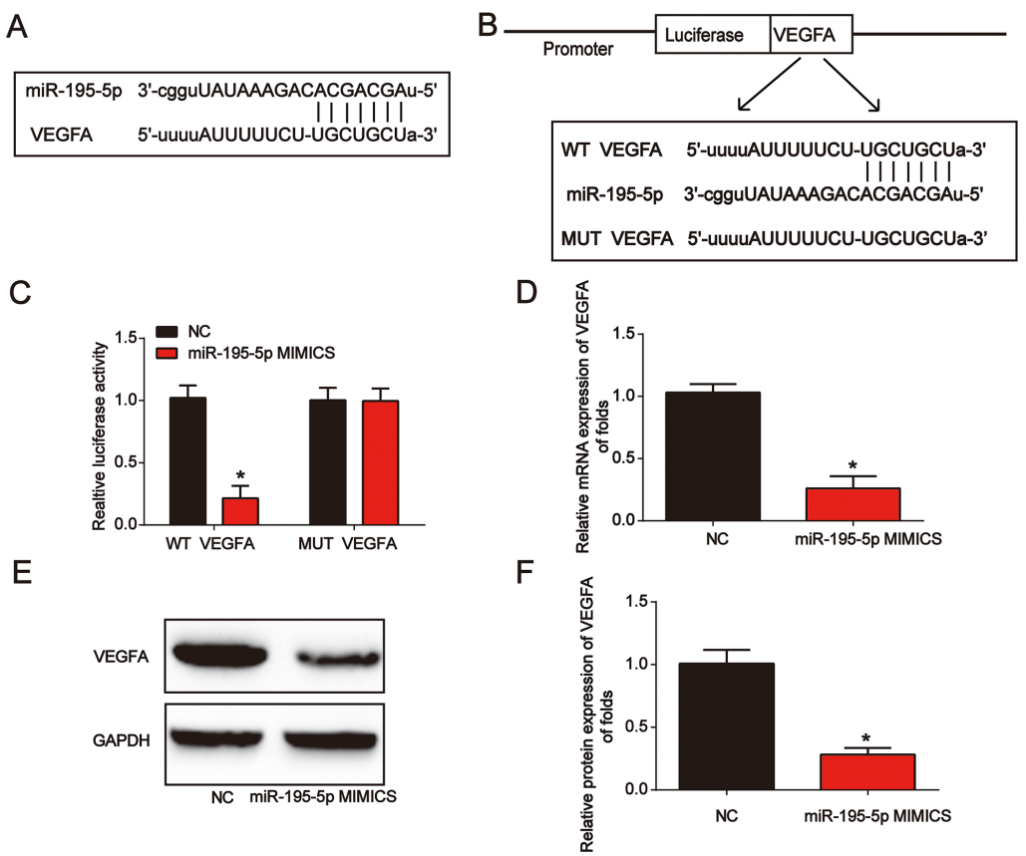
**VEGFA directly targeted miR-195-5p**. (**A**) The binding correlation between miR-195-5p and VEGFA. (**B**) Luciferase reporter constructs containing the WT-VEGFA or MUT-VEGFA sequence. (**C**) WT-VEGFA or MUT-VEGFA was co-transfected into NRK-52E cells with miR-195-5p mimics or their corresponding negative controls. (**D**) VEGFA mRNA expression in NRK-52E cells transfected with miR-195-5p mimics. (**E** and **F**) VEGFA protein expression in NRK-52E cells. Three independent experiments were performed. Error bars represent the mean ± SD of at least three independent experiments. *P < 0.05.

Next, VEGFA was predicted as the target of miR-195-5p. In Figure 5A, the binding regions between them were manifested. Luciferase reporter plasmids of WT-VEGFA and MUT-VEGFA are shown in Figure 5B. Subsequently, we observed that co-transfection of WT-VEGFA with miR-195-5p mimics suppressed the reporter activity in NRK-52E cells (Figure 5C). miR-195-5p mimics greatly inhibited VEGFA expression in NRK-52E cells (Figure 5D–5F). These results showed that miR-195-5p directly targeted VEGFA.

### VEGFA expression was upregulated in AKI

**Figure 6:**
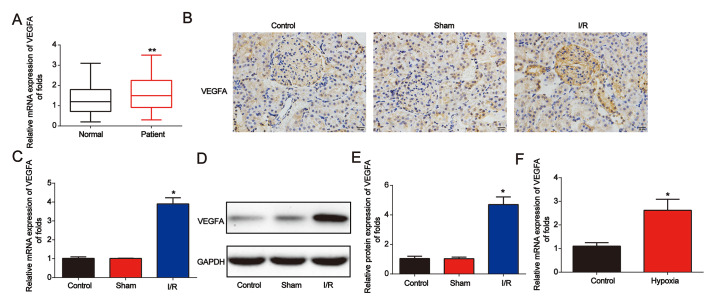
**VEGFA expression was downregulated in AKI**. (**A**) Analysis of VEGFA mRNA expression in serum from healthy controls and patients with AKI. GAPDH served as a reference control. (**B**) Immunohistochemical staining of VEGFA in kidney tissues from I/R rats. (**C–E**) VEGFA expression in I/R rat models. (**F**) VEGFA mRNA expression in NRK-52E cells exposed to hypoxia for 6 h. Eight rats were used in each group. Three independent experiments were performed. Error bars represent the mean ± SD of at least three independent experiments. *P < 0.05; **P < 0.01.

Next, we found that VEGFA expression was significantly increased in AKI patients (Figure 6A). Then, in Figure 6B, in the renal I/R rat model, IHC staining indicated that VEGFA was greatly increased (Figure 6B). In addition, as shown in Figure 6C–6E, VEGFA mRNA and protein levels were upregulated. VEGFA was also induced by hypoxia in NRK-52E cells (Figure 6F). These data indicated that VEGFA was overexpressed in AKI.

### Knockdown of VEGFA repressed renal injury induced by miR-195-5p inhibitors under hypoxic conditions in vitro

**Figure 7:**
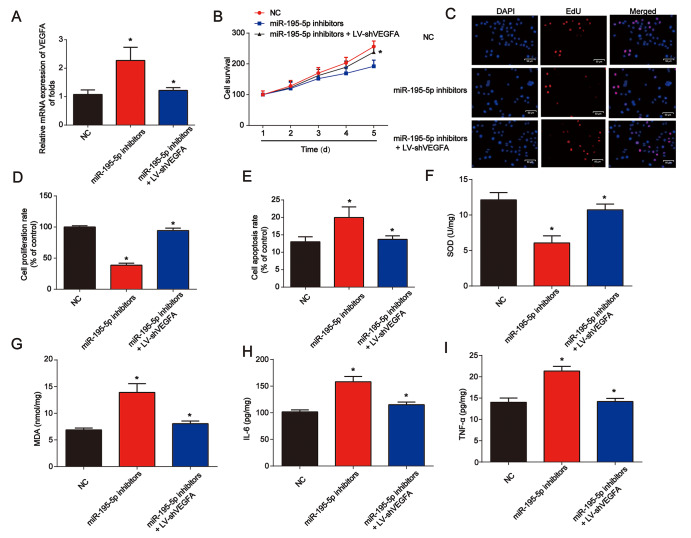
**Loss of VEGFA repressed renal injury in vitro and in vivo**. (**A**) VEGFA mRNA expression. NRK-52E cells were transfected with miR-195-5p inhibitors and infected with LV-shVEGFA under hypoxic conditions. (**B**) The CCK-8 assay was used to evaluate cell survival. (**C** and **D**) EdU assay was performed to detect cell proliferation. (**E**) Flow cytometry was used to detect cell apoptosis. miR-195-5p inhibitors and LV-shVEGFA were injected into the rat via the tail vein prior to I/R surgery. SOD (**F**) and MDA (**G**) were evaluated in kidney tissues. IL-6 (**H**) and TNF-α (**I**) protein levels in kidney tissues from rats. Eight rats were used in each group. Three independent experiments were performed. Error bars represent the mean ± SD of at least three independent experiments. *P < 0.05.

In Figure 7A, NRK-52E cells were exposed to hypoxic conditions for 6 h. Cells were then transfected with miR-195-5p inhibitors for 48 h before infection by LV-shVEGFA. We found that VEGFA was successfully repressed by LV-shVEGFA. The CCK-8 assay showed that miR-195-5p-induced cell survival was inhibited by the loss of VEGFA (Figure 7B). In Figure 7C and 7D, the EdU assay showed that NRK-52E cell proliferation was also repressed by the knockdown of VEGFA. In addition, NRK-52E cell apoptosis was reduced by the loss of VEGFA (Figure 7E). miR-195-5p inhibitors and LV-shVEGFA were injected into the rat via the tail vein. We found that SOD was inhibited by miR-195-5p inhibitors, which could be reversed by VEGFA shRNA in vivo (Figure 7F). MDA content in the kidney tissues was increased by miR-195-5p inhibitors, while loss of VEGFA was reduced (Figure 7G). In addition, inflammation factors IL-6 and TNF-α protein levels were also markedly repressed by the inhibition of VEGFA, as shown in Figure 7H and 7I.

## DISCUSSION

Recently, a growing number of microRNAs have been reported in AKI [19–21]. For example, urinary miR-30c-5p and miR-192-5p can function as biomarkers of I/R-induced kidney injury [22]. miR-709 can regulate acute tubular injury by regulating mitochondrial function [23]. miR-140-5p can reduce oxidative stress in AKI by activating Nrf2/ARE [24].

Here, we investigated the biological role of miR-195-5p in AKI. We reported that miR-195-5p expression in AKI patients, I/R rat models, and hypoxia-incubated NRK-52E cells was significantly decreased. Overexpression of miR-195-5p improved NRK-52E cell proliferation and reduced apoptosis induced by hypoxia. miR-195-5p alleviated renal injury induced by I/R in vivo As indicated, miR-195-5p mimics attenuated renal I/R-induced oxidative stress and repressed the protein expression of inflammatory factors. Moreover, VEGFA was predicted to be the target for miR-195-5p. Knockdown of VEGFA induced NRK-52E cell proliferation and inhibited apoptosis triggered by the inhibitors of miR-195-5p.

miR-195-5p is involved in the REGγ-regulated Wnt pathway in RCC [25]. miR-195 can induce mouse podocyte apoptosis by activating caspase activity [26]. Downregulated miR-195 protects mesangial cells from apoptosis in mice [27]. miR-195-5p levels were decreased in AKI patients. miR-195-5p markedly inhibited renal injury Additionally, inflammation and oxidative stress were markedly repressed by miR-195-5p overexpression. Moreover, ceRNA interaction has been widely investigated in recent years. For example, lncRNA BANCR can inhibit pancreatic cancer progression by modulating miR-195-5p [28]. Loss of lncRNA SNHG12 could reverse IGF1R-triggered osteosarcoma progression by targeting miR-195-5p [29]. Therefore, other potential mechanisms might be involved upstream of miR-195-5p in AKI. In our future study, we would like to focus on whether some lncRNAs could regulate miR-195-5p to modulate AKI.

A pathogenetic role for VEGF has been shown in the development of diabetic kidney disease [30]. VEGFA accumulates in the glomerular basement membrane during podocyte injury [31]. miR-185 can inhibit cell proliferation and increase apoptosis by regulating VEGFA in clear cell renal cell carcinoma [32]. We found that VEGFA was significantly increased in AKI patients. In addition, VEGFA was predicted as a target for miR-195-5p, and it was negatively regulated by miR-195-5p. The loss of VEGFA repressed renal injury by reducing inflammation and oxidative stress, which was induced by miR-195-5p inhibitors. In our future study, we will investigate whether other mRNA transcripts could be downstream targets for miR-195-5p.

In conclusion, miR-195-5p protected against AKI by repressing inflammation and oxidative stress by targeting VEGFA. This indicates that miR-195-5p might function as a biomarker for AKI.

## MATERIALS AND METHODS

### Patient samples

Blood samples from healthy controls and patients with AKI were obtained from the Department of Nephrology, Huai'an Second People's Hospital and the Affiliated Huai'an Hospital of Xuzhou Medical University, Huai'an, China. The study was approved by the Institutional Ethics Committee of Huai’an Second People's Hospital and the Affiliated Huai’an Hospital of Xuzhou Medical University. The research was performed according to government policies and the Helsinki Declaration. All participants provided written informed consent.

### Cell culture

NRK-52E cells were purchased from ATCC (Manassas, VA, USA). Cells were then maintained in an incubator with 5% CO_2_ at 37 °C in DMEM with 10% FBS (Gibco, Gaithersburg, MD, USA). For hypoxia assays, cells were incubated in a hypoxic chamber (Mitsubishi Gas Chemical Co., Inc., Tokyo, Japan) with 1% O_2_ for 6 h to generate hypoxia cell models.

### Animals

Eight-week-old female Wistar rats (260-280 g) were obtained from Shanghai Animal Laboratory Center). To perform renal I/R surgery, the rats were anesthetized using 50 mg/kg sodium pentobarbital. Midline laparotomy was used to expose the kidneys, and nontraumatic microaneurysm clamps were utilized to expose the renal pedicle closure for 30 min. Then, we released the microaneurysm clamps. After we observed the reperfusion, the abdomen was sealed. Before I/R surgery, miR-195-5p mimics, miR-195-5p inhibitors, LV-shVEGFA, or the negative controls were delivered into the rat via the tail vein. Eight rats were used in each group. Rats were kept according to the Guide for the Care and Use of Laboratory Animals of the National Academy of Sciences. All experiments were approved and supervised by the Animal Welfare and Ethics Committee of Huai ‘an Second People's Hospital and the Affiliated Huai’ an Hospital of Xuzhou Medical University.

### HE staining of kidney tissues

Paraformaldehyde (4%) was used to fix the kidneys. Then, the fixed kidney tissues were dehydrated, embedded in paraffin wax, and sectioned at 2 μm thickness. Afterward, the sections were de-waxed, rehydrated, and stained by H&E staining.

### Biochemical measurements of serum samples

Blood samples from the rats were kept in heparinized tubes. BUN and Cr levels were determined using the corresponding detection kits (Nanjing Jian Cheng Institute of Biotechnology, Nanjing, China).

### Immunohistochemical staining

Sections from the rats of 4 μm thickness were mounted and the slides were deparaffinized and rehydrated. To pretreat the sections, citrate buffer was used for 20 min. Then, sections were immersed in PBS with 3% H_2_O_2_. Rabbit polyclonal anti-VEGFA (dilution 1:100) was used to incubate the sections at 4°C overnight. Goat anti-rabbit IgG was then used, and the sections were treated with 3,30-diaminobenzidine chromogen.

### Cell transfection

miR-195-5p mimics (50 nmol) and miR-195-5p inhibitors (100 nmol) and their corresponding NCs were obtained from GenePharma (Shanghai, China) and were transfected using Lipofectamine 3000 (Invitrogen, Carlsbad, CA, USA). Lentivirus particles expressing sh-VEGFA were obtained from GenePharma (Shanghai, China).

### CCK-8 assay

A CCK8 kit (Beyotime, Shanghai) was used to test cell survival. Cells were plated in 96-well plates. Additionally, CCK8 reagent was added to the cells for 4 h. A microplate reader was used to measure the absorbance at 490 nm.

### EdU assay

An EdU detection kit was employed to test cell proliferation. EdU (50 μM) was added for 2 h. The cells were then fixed using 4% paraformaldehyde for half an hour. Anti-EdU working reagents were used to label the cells. Then, Hoechst 33342 was used to stain the cells. Fluorescence microscopy was utilized.

### Cell apoptosis assay

An Apoptosis Detection Kit II (BD Biosciences, San Jose, CA, USA) was used to determine cell apoptosis. First, cells were re-suspended using binding buffer. Annexin V-FITC (2 μL) was used to stain the cells. Then, 400 μL PBS and 1 μL PI were added. In order to observe the apoptotic cells, Flow cytometry (BD Biosciences, San Jose, CA) was utilized.

### TUNEL apoptosis assay

A TUNEL detection kit (Promega, Madison, WI, USA) was used to test cell apoptosis. After the tissues were paraffin-embedded, deparaffinized, and dehydrated, 20 μg/mL Proteinase K was used to incubate the sections, and the sections were rinsed using 0.3% Triton X-100. A TUNEL reaction mixture was added. Then, HRP-conjugated streptavidin was applied for half an hour. DAB (0.04%) and H_2_O_2_ (0.03%) were used for visualization. Each section was observed using a light microscope, and we counted the cells in five randomly selected fields. Apoptotic cell number/total cell number was used to calculate the apoptotic index.

### qRT-PCR

Total RNA was extracted using Trizol reagent (Invitrogen, Carlsbad, CA, USA). Then, RNA was reverse-transcribed into cDNA using a reverse cDNA synthesis kit (Takara, Shiga, Japan). SYBR-Green qPCR mix (Toyobo, Osaka, Japan) was used on the ABI 7900 Fast Real-time PCR system. Gene expression was calculated using 2^−ΔΔCt^. The primers are listed in Table 1.

**Table 1:** Primers used for real-time PCR.

**Genes**	**Forward (5’-3’)**	**Reverse (5’-3’)**
**GAPDH**	CAAGGTCATCCATGACAACTTTG	GTCCACCACCCTGTTGCTGTAG
**U6**	CTCGCTTCGGCAGCACA	AACGCTTCACGAATTTGCGT
**VEGFA**	TCACCAAGGCCAGCACATAG	GAGGCTCCAGGGCATTAGA
**miR-195-5p**	GGGGTA GCAGCACAGAAAT	TCCAGTGC GTGTCGTGGA

### Western blot

Proteins were electrophoresed on a 10% SDS-polyacrylamide gel. Then, to transfer the proteins, a PVDF membrane (Millipore, Billerica, MA, USA) was employed. The membrane was incubated in primary antibodies for a whole night. The next day, the membranes were incubated with the secondary antibodies. An ECL kit (P0018, Beyotime, Beijing, China) was used to visualize the membranes.

### Bioinformatics analysis

TargetScan 7.0 (http://www.targetscan.org/) and Starbase (http://starbase.sysu.edu.cn/) were carried out to predict VEGFA as putative targets of miR-195-5p.

### Luciferase assay

The 3’-UTR of VEGFA with miR-195-5p binding sites was inserted into a pMIR-REPORT vector. Cells were co-transfected with miR-195-5p mimics, pMIR-VEGFA 3′-UTR wild-type vector or pMIR-VEGFA 3′-UTR mutant vector. Dual-Luciferase Reporter Assay System (Promega, Madison, WI, USA) was used.

### Statistical analysis

Statistical analysis was carried out using SPSS 22.0 software. Statistical differences among different groups were analyzed using one-way ANOVA and Student’s t-test. Significance was considered when the P value was less than 0.05.
